# Identifying Family and Unpaid Caregivers in Electronic Health Records: Descriptive Analysis

**DOI:** 10.2196/35623

**Published:** 2022-07-18

**Authors:** Jessica E Ma, Janet Grubber, Cynthia J Coffman, Virginia Wang, S Nicole Hastings, Kelli D Allen, Megan Shepherd-Banigan, Kasey Decosimo, Joshua Dadolf, Caitlin Sullivan, Nina R Sperber, Courtney H Van Houtven

**Affiliations:** 1 Geriatric Research, Education, and Clinical Center Durham Veterans Affairs Health Care System Durham, NC United States; 2 Division of General Internal Medicine Department of Medicine Duke University School of Medicine Durham, NC United States; 3 Center of Innovation to Accelerate Discovery and Practice Transformation Durham Veterans Affairs Health Care System Durham, NC United States; 4 Department of Biostatistics and Bioinformatics Duke University Medical Center Durham, NC United States; 5 Department of Population Health Sciences Duke University Durham, NC United States; 6 Duke-Margolis Center for Health Policy Duke University Durham, NC United States; 7 Center for the Study of Aging Duke University School of Medicine Durham, NC United States; 8 Division of Geriatrics Department of Medicine Duke University School of Medicine Durham, NC United States; 9 Department of Medicine University of North Carolina at Chapel Hill Chapel Hill, NC United States; 10 Thurston Arthritis Research Center University of North Carolina at Chapel Hill Chapel Hill, NC United States

**Keywords:** veterans, caregivers, electronic health record

## Abstract

**Background:**

Most efforts to identify caregivers for research use passive approaches such as self-nomination. We describe an approach in which electronic health records (EHRs) can help identify, recruit, and increase diverse representations of family and other unpaid caregivers.

**Objective:**

Few health systems have implemented systematic processes for identifying caregivers. This study aimed to develop and evaluate an EHR-driven process for identifying veterans likely to have unpaid caregivers in a caregiver survey study. We additionally examined whether there were EHR-derived veteran characteristics associated with veterans having unpaid caregivers.

**Methods:**

We selected EHR home- and community-based referrals suggestive of veterans’ need for supportive care from friends or family. We identified veterans with these referrals across the 8 US Department of Veteran Affairs medical centers enrolled in our study. Phone calls to a subset of these veterans confirmed whether they had a caregiver, specifically an unpaid caregiver. We calculated the screening contact rate for unpaid caregivers of veterans using attempted phone screening and for those who completed phone screening. The veteran characteristics from the EHR were compared across referral and screening groups using descriptive statistics, and logistic regression was used to compare the likelihood of having an unpaid caregiver among veterans who completed phone screening.

**Results:**

During the study period, our EHR-driven process identified 12,212 veterans with home- and community-based referrals; 2134 (17.47%) veteran households were called for phone screening. Among the 2134 veterans called, 1367 (64.06%) answered the call, and 813 (38.1%) veterans had a caregiver based on self-report of the veteran, their caregiver, or another person in the household. The unpaid caregiver identification rate was 38.1% and 59.5% among those with an attempted phone screening and completed phone screening, respectively. Veterans had increased odds of having an unpaid caregiver if they were married (adjusted odds ratio [OR] 2.69, 95% CI 1.68-4.34), had respite care (adjusted OR 2.17, 95% CI 1.41-3.41), or had adult day health care (adjusted OR 3.69, 95% CI 1.60-10.00). Veterans with a dementia diagnosis (adjusted OR 1.37, 95% CI 1.00-1.89) or veteran-directed care referral (adjusted OR 1.95, 95% CI 0.97-4.20) were also suggestive of an association with having an unpaid caregiver.

**Conclusions:**

The EHR-driven process to identify veterans likely to have unpaid caregivers is systematic and resource intensive. Approximately 60% (813/1367) of veterans who were successfully screened had unpaid caregivers. In the absence of discrete fields in the EHR, our EHR-driven process can be used to identify unpaid caregivers; however, incorporating caregiver identification fields into the EHR would support a more efficient and systematic identification of caregivers.

**Trial Registration:**

ClincalTrials.gov NCT03474380; https://clinicaltrials.gov/ct2/show/NCT03474380

## Introduction

In the United States, approximately 26.4 million people provide unpaid care to adults aged >50 years [1]. Family and other unpaid caregivers (hereafter called unpaid caregivers) are people who provide volunteer care to a loved one [2]. The care provided by unpaid caregivers is associated with reduced hospital readmissions and increased time at home for patients [3-5]. At the same time, unpaid caregivers are at risk of negative impacts on their physical, social, emotional, and financial well-being. Most unpaid caregivers provide care with little or no training or support [6-11]. Furthermore, unless present for appointments or enrolled in caregiver programs, caregivers are not easily identified within the health systems.

To address the unmet needs of unpaid caregivers, outreach is a critical step. However, there is no systematic method of identifying caregivers for supportive services or research interventions. Prior caregiver research studies relied on caregivers to respond to advertisements or through existing caregiver programs [12-16]. Past approaches may not be ideal for overburdened caregivers, especially for those who are not already involved in these programs, and could result in underwhelming caregiver response and participation. Moreover, passive approaches can decrease representativeness and bias toward caregivers already engaged in these interventions or those already empowered to actively seek help or services [17].

Given the need to improve the representation and systematic identification of unpaid caregivers for caregiving research and supportive services, a systematic, reliable, and proactive process to identify caregivers is needed to increase engagement, expand participation, and reduce sample bias. The electronic health record (EHR) provides an important standardized method of identifying and reaching potential caregivers for research and interventions. The aim of this paper was to evaluate an EHR-driven process used to identify unpaid caregivers for a caregiver survey study in the US Department of Veteran Affairs (VA) cluster-randomized, multisite, stepped-wedge, pragmatic trial—implementation of Helping Invested Families Improve Veteran Experiences Study (iHI-FIVES) [18]. We describe the patient-focused EHR-driven process, its caregiver identification capabilities, and veteran characteristics across the initial EHR and screening groups.

## Methods

This study specifically analyzed caregiver identification through patient EHRs for a caregiver survey from the overarching iHI-FIVES study [19].

### Setting

iHI-FIVES was conducted at 8 VA medical centers from April 2018 to October 2020 using a type III hybrid implementation effectiveness stepped-wedge, cluster-randomized trial design as part of the Optimizing Function and Independence Quality Enhancement Research Initiative program. The study evaluated the implementation of an unpaid caregiver program designed to promote the function and independence of veterans through caregiver group training aimed at improving caregiver coping, support seeking, and health system navigation skills [18,20]. Eligible caregivers were friends or family members who assisted a patient at home because of ongoing health problems (eg, helping them get around the house, bathe, or pay bills) [19]. This study focused on the identification of caregivers for a caregiver survey that was administered at all sites to assess secondary outcomes (caregiver burden, depression, and satisfaction with VA care).

### Ethics Approval

The iHI-FIVES study was approved by the Durham VA Health Care System institutional review board (02040) and registered at ClinicalTrials.gov (NCT03474380).

### EHR-Driven Process

As there is no standardized method in EHRs for identifying patients with unpaid caregivers, we evaluated an EHR-driven process to identify unpaid caregivers and patient characteristics (eg, age, race, ethnicity, sex, and comorbidities) associated with having an unpaid caregiver.

We identified 5 relevant VA home and community-based services as these 5 referral types represent an increased need for care in the veteran’s home and are likely to indicate the presence of caregivers [21]. These 5 referral types included homemaker home health care, home-based primary care, adult day health care, respite care, and veteran-directed care. Homemaker home health care provides assistance in daily activities (eg, eating, getting dressed, grooming, bathing, and going to the bathroom) through an aide from a home health care agency. Adult day health care is a full- or half-day program for veterans to be supervised for daily activities during the weekday. Home-based primary care provides veterans who have difficulty traveling to clinic visits to their illness with primary care, social work, and rehabilitation visits at home. Respite care provides home or nursing home care when a caregiver is unavailable. Veteran-directed care assists veterans in connecting to community care services for daily activities [21].

There is no uniform referral naming convention for these 5 categories of home and community-based services in the VA; therefore, we purposively selected related keywords in the text of EHR referral (eg, *%home%*, *%respit%*, *%grec%*, *%adult%*, *%adhc%*, *%hbpc%*, *%vd hcbs%*, and *%veteran directed%*). The study team reviewed referral titles identified by the keywords and then applied exclusionary conditions to the programmed code to weed out inappropriate referrals garnered by the broad search. The culled list of referral titles was also confirmed by clinical experts (physicians and caregiver support coordinators) and VA EHR programmers (Multimedia Appendix 1). Some enrolled sites requested expansion beyond these 5 referral types because of the high likelihood of having an active unpaid caregiver present (eg, skilled home health referrals). We subsequently included all these referrals, as well as one additional (respiratory therapy for chronic obstructive pulmonary disease inpatient home transition program) referral in our EHR-driven process, classifying them as *other referrals*.

### Screening

We used the EHR-driven process to identify all veterans in our study window who had these qualifying referral types. The study team identified veteran patients with a qualifying referral by using VA EHR data stored in the VA Corporate Data Warehouse. Patients with referrals to hospice care were excluded [19].

Owing to the limited contact with veterans and an option to opt out, waivers of informed consent and Health Insurance Portability and Accountability Act documentation were approved for research contact with patients in this study. As the study focused on caregivers and institutional review board approval, the study team could directly confirm and seek nonveterans’ participation in the screening process and study. In addition, veterans’ treating clinicians were not involved in the identification process.

Identified veterans were stratified by site, sorted by the earliest qualifying referral date, and selected for recruitment over a 30-month study period. Those with the earliest dates were mailed a recruitment letter with an opt-out telephone number. The letter described that if a family member or friend helps the veteran with their ongoing health problem, they may qualify for a caregiver study. As the goal of the iHI-FIVES caregiver survey study was to enroll and collect baseline surveys for approximately 450 caregiver surveys, the study staff conducted phone screening until caregiver recruitment goals were met at each site.

For the telephone screening, the study staff used the veteran’s phone number and assessed the presence of a caregiver based on information provided by the veteran, the caregiver, or another person(s) who answered the phone if neither the veteran nor the caregiver was available at the time of the call. As it is not always the case that veterans or caregivers recognize the term *caregiver* or do not identify as having or being a caregiver, the screener was written to identify assistance. Specifically, veterans were asked, “Do you have a family member or friend who helps care for you because of your ongoing health problems (for example, helping you get around the house, bathe, or pay bills?).” If nonveterans answered the phone, a similar screening question was asked, starting with “Does the Veteran...” The screening script distinguished between paid, formally trained caregivers such as home health aides and caregivers who were not paid typically and for whom the veteran had an established personal relationship (eg, friends, family). The script also asked if the veteran did not have a caregiver and whether they needed help with their care.

The study team used 2 call attempts to contact veterans after sending the letter to determine if they had a caregiver. For phone messages, the study team used a general Health Insurance Portability and Accountability Act–compliant script describing a VA research study on friends or family who help care for a veteran and a follow-up phone call attempt.

The study team used DatStat Illume (version 6.1) [22] to administer and store phone screens and baseline and 3-month survey data. In the event of a highly distressed caregiver respondent, the study staff was trained in administering a harm protocol.

### EHR Data and Measures

**Figure 1:**
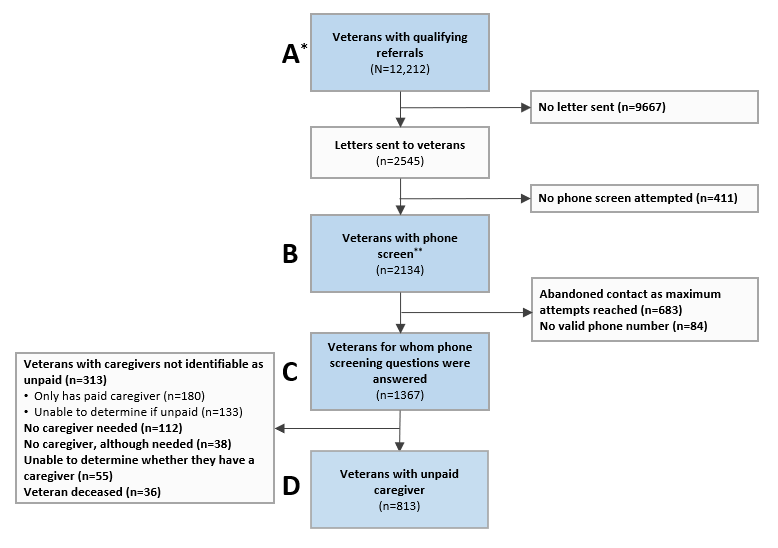
CONSORT (Consolidated Standards of Reporting Trials) diagram for the iHI-FIVES (implementation of Helping Invested Families Improve Veteran Experiences Study). *The blue callouts (letters A to D) indicate the columns listed in Table 1. **The phone screen may have involved speaking to only the veteran, only the caregiver, or both the veteran and the caregiver, which may have occurred during only one call or across multiple calls.

This study also evaluated whether particular veteran characteristics were associated with veterans having an unpaid caregiver. To examine these questions, we compared four patient groups: (1) patients with a referral to home- and community-based services, (2) patients contacted for a phone screen, (3) patients with a completed phone screen, and (4) patients confirmed through screening to have an unpaid caregiver (we label these groups as boxes A-D in the CONSORT [Consolidated Standards of Reporting Trials] diagram, Figure 1).

The study identified the following patient data from the VA Corporate Data Warehouse: demographics (age, race, ethnicity, marital status, and rural residence), VA health care eligibility (measured by service connection), outpatient International Classification of Diseases–10th Revision (ICD-10) codes in the prior year, chronic health condition risk score measuring expected health care costs compared with the average patient (Nosos [23]), and home- and community-based care referrals. We used ICD-10 codes to group patient comorbidities by Quan Deyo diagnosis categories [24] to identify patients with dementia and calculate the Charlson Comorbidity Index (CCI) [24,25]. We only included dementia, as patients with dementia have known increased caregiver needs [26]. Nosos risk scores were centered around 1, representing the national expected average cost of VA patients. A risk score >1 represents a higher than expected cost for the patient, whereas a score of 3 represents a patient with an expected cost 3 times higher than the average patient [23,27]. VA service connection indicates a medical condition associated with a veteran’s military service, for which the VA completely subsidizes all health care costs [28].

### Statistical Analyses

We calculated the screening contact rate based on the study by Slattery et al [29]. In addition, we calculated the unpaid caregiver identification rates. The screening contact rate is the percentage of veterans for whom our study staff was able to talk to a person (veteran, their caregiver, or another person(s)) when we called the veterans’ phone number of record, out of all phone screening calls attempted by research staff (see box B in the CONSORT diagram in Figure 1). The unpaid caregiver identification rate is the number of veterans positively identified to have an unpaid caregiver divided by the denominator of interest (phone screen attempted [box B] and phone screen questions completed [box C]; see the CONSORT diagram in Figure 1).

Descriptive statistics for the veteran characteristics are presented for the 4 subgroups described previously. Descriptive statistics were calculated for dichotomous (sex, ethnicity, rural residence, service connectedness, and dementia by ICD-10 codes), categorical (race, marital status, rural residence, highest level of education, and qualifying referral type), and continuous (age, CCI, and Nosos score) variables. Percentages were calculated for dichotomous and categorical data and means and SDs or medians and IQRs for continuous variables.

Simple logistic regression models were fit to estimate the association (odds ratio [OR] and 95% CI) between having an unpaid caregiver (vs not having one; among veterans with a completed phone screen) for each demographic and clinical variable defined previously. A multivariable logistic model was then fit with the same dependent variable but with all demographic and clinical variables examined independently in the simple models into one model to examine adjusted associations. Complete EHR data were used for the veteran factors in the analyses. The reference groups for the analyses were age (<55 years), sex (female), race (White), ethnicity (not Hispanic), marital status (never married), rural residence (urban), service connected (not service connected), dementia (absence of dementia diagnosis), CCI (score of ≤2), Nosos (score of ≤1), and referral qualification (homemaker home health care). The purpose of these models was to determine whether any veteran demographics, disease burden, health care cost risk scores, or referral types were associated with having an unpaid character with the idea that if such associations exist, they might inform attempts to recruit caregivers into training or research studies.

Analyses were performed using R (version 4.0.3) [30].

## Results

### Descriptives of Veteran Groups

A total of 12,212 veterans (box A in Figure 1) were identified through the EHR-driven process of 5 home- and community-based service referrals. A subset of veterans (2545/12,212, 20.84%) was sent a letter describing the study, followed by attempts to call in order of earliest qualifying referral. Only one-fifth were sent letters and screened by phone because of recruitment goals and resource limitations. Of the 2545 veterans who were sent a letter, 2134 (83.94%; box B in Figure 1) had an attempted phone screening. Of the 2545 veterans, no phone call was attempted for 411 (16.15%); 34 of those opted out of the study prior to a phone call attempt. Of the 2134 veterans contacted for phone screening, 1367 (64.06%; box C in Figure 1) veterans were reached, and 767 (35.94%) were unable to be reached. Therefore, the screening contact rate for initial veteran screening for unpaid caregivers was 64.06% (1367/2134).

Of the 1367 veterans who completed the screening questions, 1126 (82.37%) had a caregiver; 813 (59.47%; box D in Figure 1) had an unpaid caregiver, and 180 (13.17%) were paid. We were unable to determine whether 11.81% (133/1126) of caregivers were unpaid or paid. The identification rates of unpaid caregivers were 38.10% (813/2134) and 59.47% (813/1367) among veterans with attempted phone screens and veterans with completed phone screens, respectively.

### Descriptive Comparisons Between Veteran Groups

The veteran characteristics were similar among the 4 groups, including age, sex, race, urban residence, CCI, and Nosos score. The most common type of referral for veterans across all subgroups of interest was a homemaker home health care referral (Table 1). In addition, as of July 7, 2021, 38 months after the start of the study, one-third (4089/12,212, 33.48%) of the identified veterans had died.

**Table 1:** Electronic health record–based demographic characteristics of veterans by study milestone^a^ (N=12,212).

Veteran characteristic		Veterans with qualifying referral (box A^b^; N=12,212)	Veterans with attempted phone screen (box B; n=2134)	Veterans with completed phone screen (box C; n=1367)	Veterans with unpaid caregiver (box D; n=813)
Age (years), mean (SD)		74.85 (11.95)	74.95 (11.85)	75.52 (11.57)	76.0 (11.9)
**Sex (male), n (%)**		11,443 (93.70)	2000 (93.72)	1285 (94.00)	772 (95.0)
	Missing	1 (0.01)	0 (0.00)	0 (0.00)	0 (0.0)
**Race, n (%)**					
	White	8723 (71.42)	1529 (71.65)	986 (72.13)	578 (71.1)
	Black	2080 (17.03)	377 (17.67)	245 (17.92)	151 (18.6)
	Multiple races or other	624 (5.11)	107 (5.01)	63 (4.61)	38 (4.7)
	Missing	785 (6.43)	121 (5.67)	73 (5.34)	46 (5.7)
**Ethnicity, n (%)**					
	Hispanic	400 (3.28)	74 (3.47)	36 (2.63)	25 (3.1)
	Missing	447 (3.66)	71 (3.33)	46 (3.37)	23 (2.8)
**Marital status, n (%)**					
	Never married	994 (8.14)	176 (8.25)	103 (7.53)	44 (5.4)
	Divorced or separated	3297 (27.00)	553 (25.91)	332 (24.29)	157 (19.3)
	Married	6155 (50.40)	1105 (51.78)	741 (54.21)	509 (62.6)
	Widow	1691 (13.85)	290 (13.59)	185 (13.53)	99 (12.2)
	Missing	75 (0.61)	10 (0.47)	6 (0.44)	4 (0.5)
**Urban residence, n (%)**		7752 (63.48)	1367 (64.06)	864 (63.20)	512 (63.0)
	Missing	3 (0.02)	1 (0.05)	1 (0.07)	0 (0.0)
**Service connected, n (%)**		7050 (57.73)	1253 (58.72)	814 (59.55)	501 (61.6)
	Missing	1 (0.01)	0 (0.00)	0 (0.00)	0 (0.0)
**Dementia, n (%)**		2597 (21.27)	485 (22.73)	320 (23.41)	227 (27.9)
	Missing	26 (0.21)	3 (0.14)	2 (0.15)	1 (0.1)
**CCI,^c^ median (IQR 25th-75th percentile)**		2.00 (1.00-4.00)	2.00 (1.00-4.00)	2.00 (1.00-4.00)	2.0 (1.0-4.0)
	Missing, n (%)	26 (0.21)	3 (0.14)	2 (0.15)	1 (0.1)
**Nosos, median (IQR 25th-75th percentile)**		1.88 (0.89-3.87)	2.06 (0.97-3.99)	2.06 (0.96-3.89)	2.1 (0.9-3.9)
	Missing, n (%)	129 (1.06)	10 (0.47)	7 (0.51)	5 (0.6)
**Referral, n (%)**					
	Homemaker home health care	7699 (63.04)	1368 (64.10)	873 (63.86)	494 (60.8)
	Adult day health care	440 (3.60)	87 (4.08)	51 (3.73)	45 (5.5)
	Home-based primary care	746 (6.11)	111 (5.20)	71 (5.19)	37 (4.6)
	Respite care	929 (7.61)	221 (10.36)	159 (11.63)	122 (15.0)
	Veteran-directed care	358 (2.93)	77 (3.61)	46 (3.37)	35 (4.3)
	Other^d^	2040 (16.70)	270 (12.65)	167 (12.22)	80 (9.8)

^a^Veterans were identified by first home or community referral placed during the study time frame.

^b^Boxes on the CONSORT (Consolidated Standards of Reporting Trials) diagram (Figure 1).

^c^CCI: Charlson Comorbidity Index.

^d^Some enrolled sites requested the addition of specific referral types (eg, skilled home health, nursing home, and specialty home care) because of the high probability of an unpaid caregiver being present when these referrals occur. We subsequently included all these referrals, as well as one additional (respiratory therapy for chronic obstructive pulmonary disease inpatient home transition program) referral, in our electronic health record–driven process, classifying them as *other*.

Certain characteristics were different among the groups. Compared with veterans with a qualifying referral (box A in Figure 1) and those contacted for phone screen (box B in Figure 1), veterans who answered phone screening (box C in Figure 1) had higher proportions of veterans who were aged 75 to 84 years (396/1367, 28.97%), married (741/1367, 54.21%), and referred for respite care (159/1367, 11.63%). Veterans with unpaid caregivers (813/12,212, 6.66%; box D in Figure 1) had higher proportions of initial referrals for respite (122/813, 15%), adult day care (45/813, 5.5%), and veteran-directed care (35/813, 4.3%) than those of the other groups.

### Modeled Associations Between Veteran Characteristics and Presence of an Unpaid Caregiver

Among veterans for whom screening questions were answered and were without missing data (1261/12,212, 10.22%), the odds of having an unpaid caregiver were higher among married veterans (unadjusted OR 2.90, 95% CI 1.88-4.50) than among unmarried veterans and veterans with dementia (unadjusted OR 2.06, 95% CI 1.55-2.75) than those without dementia. Similarly, the odds of having an unpaid caregiver were higher among veterans referred for respite services (unadjusted OR 2.64, 95% CI 1.76-4.06), adult day health care (unadjusted OR 4.82, 95% CI 2.17-12.80), and veteran-directed care (unadjusted OR 2.07, 95% CI 1.05-4.38) than for veterans referred to homemaker home health care. In addition to dementia diagnosis and veteran-directed care, adjusted ORs for the associations between having an unpaid caregiver and marital status, as well as health care referral type, were similar to the unadjusted results (Table 2).

**Table 2:** ORs^a^ and 95% CIs from simple logistic regression models (unadjusted) and multiple logistic regression models (adjusted) for the association between veterans having unpaid caregivers among veterans with a completed phone screen and without missing data (N=1261)^b^ and veteran electronic health record characteristics.

Veteran characteristic			Unadjusted^c^					Adjusted^d^		
			OR (95% CI)		*P* value		OR (95% CI)			*P* value
**Age (years)**										
	<55	1.00 (reference)		N/A^e^		1.00 (reference)			N/A	
	55-64	0.78 (0.41-1.47)		.45		0.71 (0.36-1.37)			.31	
	65-74	0.71 (0.40-1.24)		.24		0.56 (0.30-1.01)			.06	
	75-84	0.96 (0.54-1.69)		.89		0.66 (0.34-1.23)			.20	
	>84	1.17 (0.65-2.08)		.60		0.80 (0.41-1.53)			.50	
**Sex**										
	Female	1.00 (reference)		N/A		1.00 (reference)			N/A	
	Male	1.49 (0.94-2.38)		.09		1.13 (0.68-1.88)			.63	
**Ethnicity**										
	Non-Hispanic	1.00 (reference)		N/A		1.00 (reference)			N/A	
	Hispanic	1.38 (0.68-2.99)		.39		0.87 (0.40-1.97)			.73	
**Race**										
	White	1.00 (reference)		N/A		1.00 (reference)			N/A	
	Black	1.14 (0.85-1.53)		.38		1.33 (0.97-1.84)			.08	
	Multiple races or other	1.17 (0.69-2.02)		.56		1.18 (0.67-2.10)			.57	
**Marital status**										
	Never married	1.00 (reference)		N/A		1.00 (reference)			N/A	
	Divorced or separated	1.17 (0.74-1.86)		.50		1.27 (0.79-2.06)			.33	
	Married	2.90 (1.88-4.50)		<.001		2.69 (1.68-4.34)			<.001	
	Widow	1.50 (0.91-2.49)		.11		1.43 (0.83-2.49)			.20	
**Rural residence**										
	Urban	1.00 (reference)		N/A		1.00 (reference)			N/A	
	Rural	1.04 (0.83-1.32)		.72		1.01 (0.78-1.30)			>.95	
**Service connection**										
	Not service connected	1.00 (reference)		N/A		1.00 (reference)			N/A	
	Service connected	1.20 (0.95-1.50)		.13		1.17 (0.90-1.51)			.24	
**Dementia**										
	No diagnosis of dementia	1.00 (reference)		N/A		1.00 (reference)			N/A	
	Dementia diagnosis	2.06 (1.55-2.75)		<.001		1.37 (1.00-1.89)			.06	
**Charlson comorbidity index**										
	≤2	1.00 (reference)		N/A		1.00 (reference)			N/A	
	>2	1.08 (0.86-1.35)		.52		1.13 (0.87-1.47)			.35	
**Nosos**										
	≤1	1.00 (reference)		N/A		1.00 (reference)			N/A	
	>1 and <4	0.87 (0.66-1.14)		.32		0.86 (0.63-1.17)			.33	
	≥4	0.91 (0.66-1.25)		.56		1.00 (0.68-1.46)			>.98	
**Referral**										
	Homemaker home health care	1.00 (reference)		N/A		1.00 (reference)			N/A	
	Adult day health care	4.82 (2.17-12.80)		<.001		3.69 (1.60-10.00)			.005	
	Home-based primary care	0.83 (0.50-1.39)		.48		0.87 (0.51-1.47)			.60	
	Respite care	2.64 (1.76-4.06)		<.001		2.17 (1.41-3.41)			<.001	
	Veteran-directed care	2.07 (1.05-4.38)		.04		1.95 (0.97-4.20)			.07	
	Other	0.69 (0.49-0.97)		.03		0.77 (0.54-1.10)			0.15	

^a^OR: odds ratio.

^b^A complete case analysis was used, and veterans with missing data (n=106) were excluded from this analysis.

^c^Unadjusted ORs from a simple logistic regression model, including a single electronic health record characteristic as an independent variable.

^d^Adjusted ORs from multiple logistic regression models, including all electronic health record characteristics.

^e^N/A: not applicable.

## Discussion

### Principal Findings

We describe an approach to systematically identify family and other unpaid caregivers in a caregiver study using the VA EHR. This EHR process was effective in identifying caregivers through EHR home- and community-based referrals.

We identified >12,000 veterans with relevant home- and community-based referrals. We called >2000 veterans for screening. Among the 1367 veterans who answered the phone screening, 813 (59.47%) had a family or other unpaid caregivers. Through the EHR-driven process, we found that veteran characteristics were similar among all 4 identified groups (veterans with qualifying referrals, attempted phone screening, completed phone screen, and unpaid caregivers); some notable exceptions included marital status, dementia, and referral type. Veterans with unpaid caregivers were more likely to be married and have a referral for adult day health care or respite care. Although not statistically significant in the adjusted analysis, dementia diagnosis and veteran-directed care referral were also suggestive of an association with having an unpaid caregiver.

### Comparison With Prior Work

This study is unique in that there are currently few replicable or standardized EHR methods for identifying veterans with unpaid caregivers. Using the EHR to identify veterans with unpaid caregivers can uniquely position the VA to identify caregivers interested in participating in research studies and increase the representation of caregivers that may not be identified through traditional avenues [31-34]. However, although EHRs can be useful for identifying patient participants through standardized fields for patient demographic, laboratory, medication, and diagnostic data, caregiver identification through EHRs can be difficult because it is rare to have a discrete caregiver field in EHRs [35].

As there is no discrete caregiver field in the VA EHR, we used selected home- and community-based referrals to define a patient population that may have an unpaid caregiver. Although this EHR-driven process was able to identify caregivers independent of their participation or nonparticipation in existing VA caregiver support and services, the process was resource intensive. To meet the goal of 450 caregiver surveys, 4 research staff members would spend approximately 8 to 16 hours every week during active data collection screening veterans or caregivers; screening approximately one-fifth of the identified EHR referral population resulted in the reaching of survey goals.

Therefore, although the EHR-driven process is a helpful approach to identifying caregivers not already directly linked to services in a health care system (and to be clear, most health systems do not have large-scale programs of caregiver support such as seen in VA), potential future improvements to the EHR should consider a discrete caregiver field to improve efficiency in identifying caregivers for research studies and interventions. VA is currently making investments to link caregiver records to veteran EHR records, beginning with those caregivers already engaged in the VA Caregiver Support Program support and services. This will allow the examination of program impacts on the outcomes of the enrolled caregivers and veterans over time.

Importantly, these caregiver EHR fields should be assessed using an activity-based brief screening question based on the types of assistance received in the home because of a patient’s health problems rather than whether a patient *has* a caregiver. The term caregiver is problematic for many older adults; many patients do not recognize that they have a caregiver, and many family members and friends do not identify themselves as caregivers [36].

This study identified several veteran characteristics associated with having an unpaid caregiver. First, we found that being married was positively associated with a veteran having an unpaid caregiver. Although marital status is rarely a variable in other EHRs or insurance claims (ie, Medicaid or Medicare) data, marital status could be a useful target for the identification of caregivers within the VA and potentially in other health systems, with certain caveats. Specifically, marital status alone may not be sufficient to determine the presence of a caregiver as the marital status may be out of date in the EHR and dependent on individual sites and personnel to update this discrete field.

Second, in the unadjusted analysis, veterans with dementia were more likely to have unpaid caregivers. The OR and 95% CI for this association were 1.37 (1.00-1.89) in the adjusted analysis, suggesting a moderate and likely clinically meaningful association. A dementia diagnosis is more likely to be in the medical record and may identify additional unpaid caregivers in the absence of information about the home care network in the EHR.

Third, focusing on homemaker home health care, respite care referrals, and veteran-directed care may also identify unpaid caregivers. Although most (494/813, 60.8%) of veterans with an unpaid caregiver had a homemaker home health care referral, veterans with respite, adult day health care, and veteran-directed care referrals were more likely to have an unpaid caregiver (122/813, 15%; 45/813, 5.5%; and 35/813, 4.3%, respectively) than veterans identified by the initial EHR pool (929/12,212, 7.61%; 440/12,212, 3.60%; and 358/12,212, 2.93%, respectively). This is likely because respite care, adult day health care, and veteran-directed care programs are specifically designed to assist unpaid caregivers who need time separate from the veterans [37,38]. As such, depending on the purpose of the study, future VA EHR-driven approaches may want to narrow the types of home- and community-based services from which to recruit unpaid caregivers, focusing on respite care, adult day health care, and veteran-directed care.

### Strengths and Limitations

A strength of this study is the creation of a systematic approach to screening a large number of veterans across multiple VA sites in the United States. Compared with a prior study at a VA medical center where 89% of patients referred to home- and community-based services reported having an unpaid caregiver, the systematic approach taken here at multiple sites with more geographic representation found that 59.47% (813/1367) of veterans had an unpaid caregiver [39]. Moreover, the percentage of unpaid caregivers may be larger, as we were unable to determine the caregiver status for 16.39% (224/1367) of the patients screened. Using geographically diverse sites, this study provides a more generalizable estimate using EHR-based, home- and community-based referrals to identify unpaid caregivers.

There are several limitations to consider. First, this analysis was limited to examining the associations between veteran characteristics and the presence of unpaid caregivers. We did not have caregiver information for veterans who were not screened or who had unpaid caregiver characteristics.

Second, the generalizability of this study outside the VA health system may be limited, as this study uses VA home- and community-based referrals that may not be available in non-VA settings. Despite this limitation, this was a multisite study, and veterans identified in the EHR were from VAs of various sizes in both urban and rural settings across many regions of the United States. Although the application of this process to identify caregivers may be limited to VA, the process described in this study may be applicable to many VA settings across the country looking to identify caregivers for research studies or for caregiver interventions. Other health care systems in the private sector with a unified EHR system that uses home- and community-based referrals could also use our approach to identify unpaid caregivers.

Finally, because of the resource-intensive process, we screened one-fifth of the veterans identified in the EHR-driven process. Even so, >1000 veterans answered phone screenings for unpaid caregivers. Even if the exact count of unpaid caregivers among all veterans identified is unattainable, a large number of veterans were successfully screened, and there was similarity in veteran characteristics across veterans with a qualifying referral and phone screen; therefore, our results may suggest a similar percentage of unpaid caregivers among veterans who were not contacted for the study.

### Conclusions

We present a systematic process for the identification of unpaid caregivers using veteran referrals and additional EHR factors for a caregiver survey study. Using this EHR-driven process, we were able to positively identify the presence of unpaid caregivers for approximately 60% (813/1367) of veterans whose households responded to phone screening calls. In the setting of a resource-intensive process, additional research is needed to increase the efficiency and effectiveness of caregiver identification efforts in EHRs for caregiver interventions and research. Potential future steps may include the incorporation of a discrete caregiver field into the EHR. This type of field would not only improve the efficiency of identifying caregivers within the EHR but also the clinical care for patients in recognizing and including the primary caregiver in the health care team.

## Appendix

Multimedia Appendix 1 SQL search term algorithm for the 5 qualifying home- and community-based services.

## Abbreviations

CCI: Charlson Comorbidity Index
CONSORT: Consolidated Standards of Reporting Trials
EHR: electronic health record
HSR&D: Health Services Research and Development
ICD-10: International Classification of Diseases–10th Revision
iHI-FIVES: implementation of Helping Invested Families Improve Veteran Experiences Study
OR: odds ratio
VA: Veterans Affairs
