# Supplementary material for: Identifying Family and Unpaid Caregivers in Electronic Health Records: Descriptive Analysis
Source: JMIR Form Res. 2022 Jul 18;6(7):e35623. doi: 10.2196/35623 (PMC9345058; doi:10.2196/35623)
Supplement: Multimedia Appendix 1 [file formative_v6i7e35623_app1.pdf]

```sql
SELECT
  [Sta3n],
  [ServiceName]

FROM [CDWWork].[Dim].[RequestService]
WHERE       (
              (servicename like '%home%' or
               servicename like '%respit%' or
               servicename like '%grec%' or
               servicename like '%adult%' or
               servicename like '%adhc%' or
               servicename like '%hbpc%' or
               servicename like '%vd hcbs%' or
               servicename like '%veteran directed%')

              and

              (servicename not like '%hospic%' and
              servicename not like '%homeles%' and
              servicename not like '%home sleep%' and
              servicename not like '%homi%' and
              servicename not like '%homestead%' and
              servicename not like '%tele%' and
              servicename not like '%zz%' and
              servicename not like '%geri%' and
              servicename not like '%mountain%' and
              servicename not like '%mt home%' and
              servicename not like '%infusion%' and
              servicename not like '%oxygen%' and
              servicename not like '%cardiac%' and
              servicename not like '%pharmacy%' and
              servicename not like '%telemental%' and
              servicename not like '%pap%' and
              servicename not like '%aerosol%' and
              servicename not like '%valve%' and
              servicename not like '%vent%' and
              servicename not like '%safety%' and
              servicename not like '%o2%' and
              servicename not like '%02%' and
              servicename not like '%suction%' and
              servicename not like '%volume%' and
              servicename not like '%prosthet%' and
              servicename not like '%foster%' and
              servicename not like '%nursing home%' and
              servicename not like '%nurs home%' and
              servicename not like '%nursinghome%' and
              servicename not like '%stride%' and
              servicename not like '%chaplain%' and
              servicename not like '%occup%' and
              servicename not like '%ot%' and
              servicename not like '%physical%' and
              servicename not like '%pt%' and
              servicename not like '%speech%' and
              servicename not like '%iv%' and
              servicename not like '%wound%' and
              servicename not like '%cognitive%' and
              servicename not like '%residential%' and
              servicename not like '%opt%' and
```

```sql
servicename not like '%care-skilled%' and
servicename not like '%grecc%' and
servicename not like '%psych%' and
servicename not like '%cvt%' and
servicename not like '%nutr%' and
servicename not like '%hospital%' and
servicename not like '%ekg%' and
servicename not like '%sleep%' and
servicename not like '% inr %' and
servicename not like '%home tube feeding instruction%' and
servicename not like '%state veterans home%' and
servicename not like '%echo%' and
servicename not like '%ipvap%' and
servicename not like '%audiology%' and
servicename not like '%blind rehab%' and
servicename not like '%lab%' and
servicename not like '%occ ther%' and
servicename not like '%cont nur home%' and
servicename not like '%dialysis%' and
servicename not like '%rehabilitation%' and
servicename not like '%eyeglass%' and
servicename not like '%anticoag%' and
servicename not like '%home bp%' and
servicename not like '%enoxaparin%' and
servicename not like '%i p v %' and
servicename not like '%occ ther%' and
servicename not like '%cont nur home%' and
servicename not like '%dialysis%' and
servicename not like '%maximyst%' and
servicename not like '%home n o d%' and
servicename not like 'home o%' and
servicename not like '%oxim%' and
servicename not like '%home ox req%' and
servicename not like '%home oxy %' and
servicename not like '%hvsep%' and
servicename not like 'hvsep%' and
servicename not like '%hyperlink to home epley handout%' and
servicename not like '% skill nursing %' and
servicename not like '%mammog%' and
servicename not like '%radiolog%' and
servicename not like '%non-formulary%' and
servicename not like 'non-formulary%' and
servicename not like '%g-home glucose%' and
servicename not like '%nsg home%' and
  (not (servicename like '%skilled%' and servicename not like
  '%non_skilled%' and servicename not like '%nonskilled%')))
)
```
